# Gonorrhoea treatment failure caused by a *Neisseria gonorrhoeae* strain with combined ceftriaxone and high-level azithromycin resistance, England, February 2018

**DOI:** 10.2807/1560-7917.ES.2018.23.27.1800323

**Published:** 2018-07-05

**Authors:** David W Eyre, Nicholas D Sanderson, Emily Lord, Natasha Regisford-Reimmer, Kevin Chau, Leanne Barker, Markus Morgan, Robert Newnham, Daniel Golparian, Magnus Unemo, Derrick W Crook, Tim EA Peto, Gwenda Hughes, Michelle J Cole, Helen Fifer, Anne Edwards, Monique I Andersson

**Affiliations:** 1 Big Data Institute, University of Oxford, Oxford, United Kingdom; 2 Nuffield Department of Medicine, University of Oxford, Oxford, United Kingdom; 3 Oxford University Hospitals NHS Foundation Trust, Oxford, United Kingdom; 4 WHO Collaborating Centre for Gonorrhoea and Other STIs, National Reference Laboratory for STIs, Örebro University Hospital, Örebro, Sweden; 5 National Infection Service, Public Health England, Colindale, United Kingdom; 6 National Institute for Health Research Health Protection Research Unit in Healthcare Associated Infections and Antimicrobial Resistance, University of Oxford, Oxford, United Kingdom; 7 The authors contributed equally to this work

**Keywords:** gonorrhoea, treatment failure, antimicrobial resistance, antimicrobial treatment, whole-genome sequencing

## Abstract

We describe a gonorrhoea case with combined high-level azithromycin resistance and ceftriaxone resistance. In February 2018, a heterosexual male was diagnosed with gonorrhoea in the United Kingdom following sexual intercourse with a locally resident female in Thailand and failed treatment with ceftriaxone plus doxycycline and subsequently spectinomycin. Resistance arose from two mechanisms combining for the first time in a genetic background similar to a commonly circulating strain. Urgent action is essential to prevent further spread.

Antimicrobial resistance in *Neisseria gonorrhoeae* is a major concern. Dual therapy with ceftriaxone and azithromycin, the last two mainstream agents to which *N. gonorrhoeae* remains largely susceptible, is widely recommended internationally [1]. We describe a case of urethral and pharyngeal infection with *N. gonorrhoeae* with combined high-level azithromycin resistance and ceftriaxone resistance. Previously no such cases have been reported.

## Case description and microbiology

In February 2018, a heterosexual male presented to a sexual health clinic in the United Kingdom (UK) with a 4-day history of urethral discharge and dysuria. He reported having had sexual intercourse 3 days earlier with a regular female partner in the UK. He also reported having had sex with a locally resident female in Thailand in January 2018; he had no history of sexually transmitted infections and no other past medical history. Examination revealed a creamy white urethral discharge, with 3 + pus cells and Gram-negative intracellular diplococci seen under microscopy, leading to a diagnosis of urethral gonorrhoea infection. He was treated with a single dose of intramuscular ceftriaxone 1 g and oral doxycycline 100 mg twice daily for 7 days.

A urine nucleic acid amplification test (NAAT) was positive for *N. gonorrhoeae* and *Chlamydia trachomatis* negative. *N. gonorrhoeae* was cultured from a urethral swab. Antimicrobial susceptibility testing was undertaken by M.I.C. Evaluator Strips (Oxoid, Basingstoke, UK), according to the manufacturer’s instructions, with results confirmed using Etest (BioMérieux, Marcy l’Etoile, France) at Public Health England, Colindale, UK and the WHO Collaborating Centre for Gonorrhoea and other STIs, Sweden. European Committee on Antimicrobial Susceptibility Testing resistance breakpoints were used [2]. The minimum inhibitory concentrations (MICs) of nine antimicrobials are given in Table 1 and demonstrate high-level resistance to azithromycin (> 256 mg/L) and resistance to ceftriaxone (0.5 mg/L), as well as tetracycline (32 mg/L) and ciprofloxacin (> 32 mg/L). The patient was recalled and during this visit, 13 days after starting ceftriaxone/doxycycline treatment, his symptoms had resolved and a urine NAAT was negative for *N. gonorrhoeae.* However, given the previous antibiotic susceptibility profile, he was treated with a single dose of intramuscular spectinomycin (2 g). At a follow-up appointment, 20 days later, a urine NAAT was negative but a pharyngeal swab (omitted accidentally at the prior visits; the patient was asymptomatic at this site) was culture-positive for *N. gonorrhoeae* thus fulfilling international criteria for a verified treatment failure [3] as no further contact was reported with the Thai female following initial treatment. The same MICs were obtained from both cultured isolates from the case. Following this, the patient was treated with ertapenem (1 g) intravenously for 3 days. Subsequent NAAT and culture of urethral and pharyngeal swabs a further 21 days later were negative. The patient’s regular UK partner had negative vaginal and pharyngeal swab NAATs. We were unable to contact the patient’s partner from Thailand who the patient had initially met via a dating website.

**Table 1:** Antimicrobial minimum inhibitory concentrations, *Neisseria gonorrhoeae* case imported from Thailand to England, February 2018

Antimicrobial	MIC	Interpretation^a^
Ceftriaxone	0.5 mg/L	Resistant
Cefixime	2 mg/L	Resistant
Azithromycin	> 256 mg/L	High-level resistant
Ciprofloxacin	> 32 mg/L	Resistant
Tetracycline	32 mg/L	Resistant
Benzylpenicillin	1 mg/L	intermediate susceptible
Spectinomycin	8 mg/L	Susceptible
Gentamicin	2 mg/L	No resistance breakpoint available (low value)
Ertapenem	0.032 mg/L	No resistance breakpoint available (low value)

MIC: minimum inhibitory concentrations.

^a^European Committee on Antimicrobial Susceptibility Testing resistance breakpoints were used [2].

## Antimicrobial resistance determinants and sequence analysis

By combining short-read (Illumina, San Diego, California, United States of America) and long-read (Oxford Nanopore Technologies, Oxford, UK) sequence data a complete hybrid 2.17Mb assembly for each isolate was determined, these have been deposited, together with raw sequence data, in the European Nucleotide Archive (PRJEB26560; see supplement for details). Mapping sequence reads to one of these novel reference genomes as previously described [4], the sequences of the pre-treatment urethral (G97687) and the post-initial treatment pharyngeal (G7944) isolates from the case were indistinguishable i.e. they were consistent with acquisition from the same source at both anatomical sites. The isolates had a novel *N. gonorrhoeae* multi-antigen sequence typing (NG-MAST) sequence type (ST), ST16848 determined *in silico* [4], and were assigned MLST 12039.

Antimicrobial resistance determinants were identified from the sequencing data as described previously using a combination of *de novo* assembly and mapping-based approaches [5]. The isolates from the case included an identical mosaic *penA* allele to that previously identified in the ceftriaxone-resistant FC428 strain isolated in Japan in 2015, which also had a ceftriaxone MIC of 0.5 mg/L [6]. This mosaic *penA* allele contains two key ceftriaxone resistance mutations, A311V and T483S, but not the T316P mutation found in the more resistant H041 strain [7]. The isolates also had the A2059G mutation in all four 23S rRNA genes – the most commonly occurring mutation responsible for high-level azithromycin resistance [8]. Additional antimicrobial resistance determinants are summarised in Table 2. The isolate had a *N. gonorrhoeae* Sequence Typing for Antimicrobial Resistance (NG-STAR) type of 996.

**Table 2:** Antimicrobial resistance determinants present in isolates, *Neisseria gonorrhoeae* case imported from Thailand to England, February 2018

Gene	Variant	Mechanism	Antimicrobials affected
23S rRNA	A2059G, 4 copies	Decreased macrolide binding to 50S ribosome	AZM
*penA*	FC428 mosaic *penA* - 100% identity	Reduced β-lactam acylation of penicillin binding protein (PBP) 2	CRO, PEN
*penB*	G120K, A121D	Reduced influx through PorB1b	CRO, PEN, TET
*mtrR*	G45D, Promoter deletion	Over-expression of MtrCDE efflux pump resulting in increased efflux	AZM, CRO, PEN, TET
*ponA*	L421P	Reduced β-lactam acylation of PBP1	PEN
*tetM*	Gene presence	Prevents tetracycline binding to the 30S ribosome	TET
*rpsJ*	V57M	Reduced affinity of 30S ribosome for tetracycline	TET
*gyrA*	S91F, D95A	Reduced quinolone binding to DNA gyrase	CIP
*parC*	S87R	Reduced quinolone binding to topoisomerase IV	CIP

AZM: azithromycin; CIP: ciprofloxacin; CRO: ceftriaxone; PEN: benzylpenicillin; TET: tetracycline.

Resistance determinants as previously described [5] were searched for. No additional *macAB*, *norM* promoter variants; *mtrR*, *pilQ* mutations; or *erm*, *mef*, *ere* genes were identified. The FC428 *penA* allele has NCBI accession number LC113953.1.

**Figure 1:**
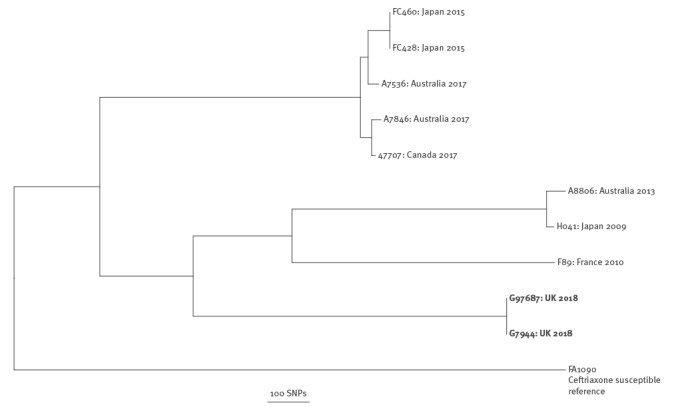
Genetic relatedness with previous ceftriaxone resistant isolates of *Neisseria gonorrhoeae* case imported from Thailand to England, February 2018

Based on reference-based mapping and correcting for recombination [4], previously sequenced ceftriaxone-resistant *N. gonorrhoeae* isolates, including the FC428 sharing the same *penA* allele, differed from our case by > 1,500 single nucleotide polymorphisms (SNPs) (Figure 1).

The figure shows a recombination-corrected maximum-likelihood phylogeny based on mapping to the *Neisseria gonorrhoeae* NCCP11945 reference genome. It shows the relationship between the case’s isolates’ sequences and previously sequenced *N. gonorrhoeae* ceftriaxone resistant isolates (from NCBI/ENA BioProjects PRJNA416507, PRJNA415047, PRJEB14020 and GenBank accession number NC_002946.2) [6,7,14-17,19,21]. Isolates from the case are in bold and labelled with the identifiers used in the text.

**Figure 2:**
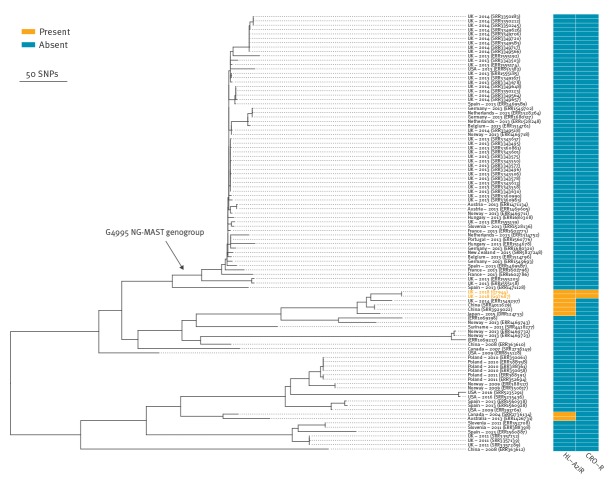
Phylogeny of the most closely related existing *Neisseria gonorrhoeae* genome sequences to the imported *Neisseria gonorrhoeae* case from Thailand to England, February 2018

CRO-R: ceftriaxone resistant; HL-AziR: high-level azithromycin resistant.

We compared our isolates’ sequences to all publicly available whole-genome sequenced *N. gonorrhoeae* isolates. There were 7,812 sequences available for comparison, including European and North American surveillance samples and samples from a global collection. Using a rapid k-mer based search (see supplement for details), we identified the 98 most closely related genomes to the two sequences identified in our case to determine a maximum-likelihood phylogeny (Figure 2). The phylogeny contains two deep clades, the top clade contains the sequences from our case, which is predominantly from NG-MAST ST4995 and closely-related STs (collectively known as genogroup G4995); our case differs from G4995 by approximately 400 SNPs. The most closely related genomes were within 45 SNPs and were obtained in China (isolation dates unavailable) with additional genomes within 54 and 71 SNPs from the UK (2014) and Japan (2015), respectively. *N. gonorrhoeae* evolves at 3.6 SNPs per genome per year [4] meaning that the most recent common ancestor of these strains is likely to be from several years earlier. These related sequences each contained four copies of the 23S rRNA A2059G mutation conferring high-level azithromycin resistance; however, all contained the ceftriaxone-susceptible *penA* II allele. No other sequence in the tree contained any of the three *penA* key mutations associated with ceftriaxone resistance i.e. A311V, T316P and T483S.

The figure shows a recombination-corrected maximum-likelihood phylogeny based on mapping to a novel hybrid assembly of the case’s sequences (ENA Sample ERS2461487). It shows the relationship between isolate sequences identified from our case and the most closely genetically related previously sequenced *Neisseria gonorrhoeae* isolates. Sequences are labelled with country and year of isolation where available (obtained either from the European Nucleotide Archive or the sequence submitter); sequence run accession numbers are provided. Antimicrobial susceptibilities were inferred from available sequence data [5]. Isolates from the case are shown in bold and labelled with the identifiers used in the text. The NG-MAST genogroup G4995’s most recent common ancestor is indicated by an arrow.

## Discussion

This is the first reported ceftriaxone-resistant, high-level azithromycin resistant *N. gonorrhoeae* isolate worldwide. The emergence of dual resistance to the last remaining mainstream treatment options poses serious challenges for the management and control of gonorrhoea infections globally. The strain was also resistant to ciprofloxacin and tetracycline. Although our case’s urethral infection was cleared with empirical ceftriaxone/doxycycline treatment, his asymptomatic pharyngeal infection failed treatment despite the relatively high dose of ceftriaxone used (1 g, compared to 250–500 mg frequently used) and the MIC just above the resistance breakpoint. This suggests that increased ceftriaxone dosing may not be adequate in similar cases. The case also failed spectinomycin treatment despite in vitro susceptibility. The limited efficacy of spectinomycin [9], as well as gentamicin [10,11] in pharyngeal infection, has been previously described.

The first sustained national transmission of isolates with high-level resistance to azithromycin (MIC ≥ 256 mg/L) has been recently documented in the UK [12], as well as a cluster of cases in Hawaii [13]. However, only a limited number of ceftriaxone-resistant isolates (MIC ≥ 0.25 mg/L) cases have been characterised in detail worldwide. These include strains H041/WHO-X (Japan 2009, ceftriaxone MIC 2–4 mg/L) [7], F89/WHO-Y (France and Spain 2010, 1–2 mg/L) [14,15], A8806/WHO-Z (Australia 2013, 0.5 mg/L) [16], GU140106 (Japan 2014, 0.5 mg/L) [17], a strain in Argentina 2014 (0.5 mg/L) [18], and FC428 (Japan 2015, 0.5 mg/L) [6]. While the earlier reports were not associated with apparent sustained onward transmission, subsequent spread of strains closely related to FC428 have been reported in Canada [19], Denmark [20] and Australia [21] in 2017 and are predominately associated with travel to south-east Asia. However, these previous strains were either susceptible, intermediate or had low-level resistance to azithromycin with MICs of 0.25–1 mg/L. Six strains in a multi-institutional series from Japan (2000–2015) [22] and several isolates from a Chinese series (2013–2016) [23] were ceftriaxone-resistant with MICs of 0.5 mg/L, as well as a case from the United States in 2017 [24]. In 2014, combined azithromycin and ceftriaxone treatment failure was reported in the UK, with MICs of 1 and 0.25 mg/L respectively [25].

Dual resistance against recommended antibiotics for first-line treatment, including high-level azithromycin resistance, had not been reported until Public Health England issued an alert regarding this current case [26]. A similar public health alert has since been released by the Australian Department of Health, whereby they reported two further cases [27].

In our case, dual resistance arose via two known antimicrobial resistance mechanisms, which combined in the same isolate for the first recorded time. Comparing our isolates with all previously genome sequenced *N. gonorrhoeae* isolates, we found that our isolates contain the same *penA* allele found in FC428 from Japan in 2015, but their overall genomes differ substantially (by > 1,500 SNPs). The isolates from our case were more closely related (circa 400 SNPs) to those from genogroup G4995, which was the eighth most commonly circulating NG-MAST genogroup in Europe in 2013 [28]. To date, the spread of ceftriaxone-resistant strains has been limited, which is possibly due to the limited fitness of these strains. However, our isolate represents the concerning new combination of a ceftriaxone resistant *penA* allele that has disseminated to several countries including Japan, Australia, Canada, and Denmark, together with high-level azithromycin resistance, in a genetic background similar to a successful and commonly circulating strain.

## Conclusion

Combined high-level resistance to azithromycin and resistance to ceftriaxone poses a global public health threat. Greater access to resistance testing, including through the development of new molecular diagnostics, is required to help guide treatment and ensure robust antimicrobial stewardship. This combined with increased use of test of cure, effective partner notification and fast-tracked development of new treatments and ideally a gonococcal vaccine, requires urgent action. The treatment failure also illustrates the severe difficulties in effectively treating pharyngeal gonorrhoea, and enhanced focus on appropriate detection and treatment of pharyngeal gonorrhoea, including in heterosexual men and women, is essential. This case, taken together with previous reports, highlights the global nature of antimicrobial resistance in gonorrhoea and in particular the importance of transmission in south-east Asia where systematic surveillance to date has been relatively limited. International cooperation is key to ensuring that gonorrhoea remains a treatable infection.
